# Real‐world brain imaging in a population‐based cohort enables accurate markers for dementia

**DOI:** 10.1002/alz.70227

**Published:** 2025-07-22

**Authors:** Reijo Sund, Juho Seppänen, Elaheh Moradi, Sami Väänänen, Jani Miettinen, Juhana Hakumäki, Toni Rikkonen, Heikki Kröger, Heli Koivumaa‐Honkanen, Alina Solomon, Jussi Tohka, for Alzheimer's Disease Neuroimaging Initiative

**Affiliations:** ^1^ Institute of Clinical Medicine University of Eastern Finland Kuopio Finland; ^2^ Knowledge Management Unit Kuopio University Hospital Kuopio Finland; ^3^ A.I. Virtanen Institute for Molecular Sciences University of Eastern Finland Kuopio Finland; ^4^ Department of Clinical Radiology Kuopio University Hospital Kuopio Finland; ^5^ Department of Technical Physics University of Eastern Finland Kuopio Finland; ^6^ Department of Orthopaedics Traumatology and Hand Surgery Kuopio University Hospital Kuopio Finland; ^7^ Ageing Epidemiology Research Unit School of Public Health Imperial College London Charing Cross Hospital London UK; ^8^ Division of Clinical Geriatrics Center for Alzheimer Research Department of Neurobiology Care Sciences and Society Karolinska Institutet Stockholm Sweden

**Keywords:** brain imaging, cognitive impairment, cognitive status, dementia, hippocampal volume, magnetic resonance imaging, memory concern, real‐world data, real‐world evidence, total gray matter volume, ventricle volume

## Abstract

**INTRODUCTION:**

Although a vast amount of magnetic resonance imaging (MRI) data are collected for health care delivery, generating real‐world evidence (RWE) in Alzheimer's disease and related dementias (ADRD) research is substantially limited by lack of methods and results showing how routine MRI scans can be used for ADRD imaging studies.

**METHODS:**

We compared three established ADRD biomarkers (total gray matter, hippocampal, and ventricular volumes) in four groups (normal, subjective complaints, mild cognitive impairment [MCI], and dementia) between the general population of women born in 1932–1941 in the Kuopio region of Eastern Finland (population‐based Kuopio Osteoporosis Risk Factor and Prevention Study [OSTPRE] cohort, *N* = 14220) and a well‐characterized research cohort (Alzheimer's Disease Neuroimaging Initiative [ADNI]).

**RESULTS:**

A total of 2434 brain MRI scans for 1885 women were collected between 2003 and 2022 by the public health care provider covering all residents in the region. The established biomarkers were overall aligned between these cohorts.

**DISCUSSION:**

Typical biomarkers extracted from real‐world brain MRI scans collected over 20 years are suitable for generating RWE in ADRD research.

**Highlights:**

- Real‐world brain magnetic resonance imaging (MRI) is applicable for generating evidence in Alzheimer's disease and related dementias (ADRD) research.
- This is the first study comparing a real‐world MRI cohort with an established research cohort reference.
- Provides a methodological framework for real‐world evidence (RWE) ADRD studies that utilize routinely collected MRI scans.

## 1 BACKGROUND

Magnetic resonance imaging (MRI) plays a crucial role in the diagnostic and prognostic evaluation of neurodegenerative diseases. Following recent regulatory approval of amyloid‐targeted disease‐modifying therapies for Alzheimer's disease (AD), MRI has also become important for assessing eligibility for treatment and monitoring the effects including adverse events.^1^ In non‐pharmacological interventions aiming to reduce the risk of dementia, MRI can provide very useful information about the potential intervention effects on the brain and the window of opportunity for prevention.^2^, ^3^ However, the number of MRI scans in clinical trials and research cohorts is limited. This limitation poses a significant challenge for developing predictive biomarkers using artificial intelligence (AI)–based methods. Furthermore, research cohorts often exhibit inherent selection biases and lack the necessary population heterogeneity.^4^, ^5^, ^6^, ^7^

In contrast, a vast amount of real‐world imaging data (RWD) is collected routinely for the delivery of health care. For instance, in the United Kingdom alone, over 700,000 brain MRI scans are performed annually.^8^ Similarly, elderly people in the United States undergo brain MRI at a rate exceeding 40 scans per 1000 persons annually.^9^ Surprisingly, the utilization of RWD in dementia research remains an underexplored area. Although some studies have focused on memory clinic populations (e.g.,^10^, ^11^, ^12^, ^13^), there exists a wealth of untapped potential in mining the picture archiving and communications systems (PACS) at hospitals.^14^

One of the key limitations in generating real‐world evidence (RWE) in Alzheimer's disease and related dementias (ADRD) research is the absence of methods and protocols as well as the lack of evidence for the potential to use routinely collected clinical MRI scans for dementia studies. The inherent difficulties associated with clinical MRI scans—such as non‐standardized pulse sequences, varied acquisition protocols, suboptimal data quality, and potential artifacts—require evidence supporting their value as imaging biomarkers. This challenge is not unique to MRI; similar obstacles persist in the broader context of medical RWD utilization.^15^

In this study, we compare ADRD biomarkers derived from routine brain MRI scans extracted from the PACS of a large regional public health care provider in Eastern Finland with a well‐established reference research cohort, Alzheimer's Disease Neuroimaging Initiative (ADNI), where imaging has followed a strict protocol. We also describe the steps needed to locate and organize RWD for MRI scans, link them to diagnostic data from medical records, and compute and quality‐check the relevant biomarkers to ensure their suitability for generating RWE in ADRD research.

## 2 METHODS

### 2.1 The OSTPRE population‐based cohort and real‐world MRI scans

The Kuopio Osteoporosis Risk Factor and Prevention Study (OSTPRE) is a population‐based cohort including all women born in 1932–1941 who lived in Kuopio County in Eastern Finland in 1989 (*N* = 14 220). The use of total population guarantees the diversity, equity, and inclusion of the target population. These women have been followed up every fifth year since 1989 using questionnaires (and measurement visits for a subpopulation). Several randomized controlled trials (RCTs) and other studies with additional data collection have also been conducted within the cohort. The OSTPRE study originally aimed to investigate factors associated with bone mineral density, bone loss, falls, and fractures, but the scope has broadened to healthy aging in general. National and local register data have been linked to the data set, including, for example, health care admissions since 1969 and clinical radiologic imaging data since 2003. The OSTPRE cohort has been described in detail previously.^16^, ^17^, ^18^, ^19^ The study has been approved by the Ethics Committee of Kuopio University Hospital. Permissions for register data have been obtained from the Finnish data permit authority for the social and health care data (Dnro THL/6840/14.02.00/2020). The study is performed in accordance with the ethical standards as laid down in the 1964 Declaration of Helsinki and its later amendments.

MRI scans are routinely stored to a regional PACS on the Wellbeing services county of North Savo, Kuopio, a large regional public health care provider in Eastern Finland. PACS started to serve Kuopio University Hospital in 2003, and district hospitals and primary care units joined the regional PACS between 2006 and 2011. Between 2003 and 2023, the regional PACS included 294,045 MRI examinations, of which 100,379 covered the head/brain region. Of the head/brain MRI examinations, 52,780 were performed for patients older than 50 years of age, and the yearly number tripled after the first years (Figure S1). Of these examinations, 44,513 were conducted in the radiology department of Kuopio University Hospital, 3448 in privately operated mobile MRI units visiting district hospitals in the neighboring towns of Iisalmi and Varkaus, and 4819 in stationary privately operated MRI units (Table S1). Most of the MRI examinations were ordered by neurology clinics (Table S2). The MRIs for the OSTPRE cohort were obtained from the regional PACS.

### 2.2 Extraction of the real‐world brain MRIs

**FIGURE 1 alz70227-fig-0001:**
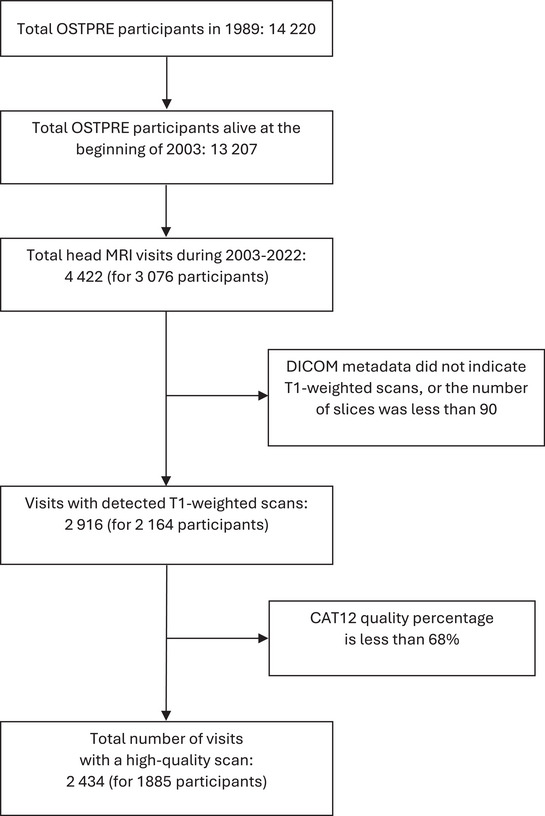
Flowchart of the real‐world brain MRI scans for OSTPRE cohort participants. MRI, magnetic resonance imaging; OSTPRE, The Kuopio Osteoporosis Risk Factor and Prevention Study.

A flowchart of the routine brain MRI scans for OSTPRE cohort participants for the years 2003–2022 is shown in Figure 1. All head area MRI examinations of the OSTPRE participants were exported from the regional PACS in which the imaging examinations were stored using the Digital Imaging and Communication in Medicine standard (DICOM, see Supplementary Text 1). First, the PACS reporting database was used to create a list of examinations of the OSTPRE participants. The list was fed to a custom script that tagged the examinations in the PACS database. The images of the tagged examinations were then sent from PACS using teleradiology to a server running storescp application from the DICOM Toolkit (version 3.6.7).^20^ A python script run by PACS before teleradiology sent pseudonymized images by erasing DICOM tags including patient identification information (e.g., name and personal identification code assigned to each Finnish resident). The server with storescp running stored the images to a network‐attached storage (NAS) device. Data were organized so that all images belonging to the same examination visit were stored in their own subfolder. These subfolders were compressed with 7‐zip and then moved to the secure research environment of the OSTPRE study. Basic metadata about the examinations were extracted from reporting databases of PACS and Radiology Information System (RIS) and from the DICOM tags of exported image files.

**RESEARCH IN CONTEXT**

1. **Systematic review**: We reviewed the literature using PubMed for original research articles comparing Alzheimer's disease and related dementias (ADRD) biomarkers derived from real‐world brain magnetic resonance imaging (MRI) scans with research cohorts using strict MRI protocols. The search phrase “real world data MRI dementia” was used. Two relevant publications identified from 48 results are appropriately cited.
2. **Interpretation**: The utilization of real‐world MRI data in ADRD research remains an underexplored area. Our findings show that typical ADRD biomarkers derived from real‐world brain MRI scans collected over 20 years are suitable for generating real‐world evidence (RWE) in dementia research.
3. **Future directions**: This study provides both the results and a methodological framework including key steps needed to locate and assemble real‐world MRI scans, identify scans of the brain versus other organs, link them to other real‐world health data, and compute and quality‐check the relevant biomarkers to ensure their suitability. Such methodological frameworks are essential to support future RWE ADRD imaging studies.

Basic metadata were used to identify MRI examinations of the head area using Finnish radiologic procedure codes (AA1BG, AA1CG, AA1DG) with[Fig alz70227-fig-0001] a structure similar to the Nordic Medico‐ Statistical Committee (NOMESCO) Classification of Surgical Procedures (NCSP).^21^, ^22^ These examinations were decompressed from the 7‐zip archives and reorganized so that each OSTPRE participant with head area MRI scans had a folder with subfolders for each separate examination visit, containing the actual 2.3 million DICOM files (separate files, not directories; in scans each slice is one file). Next, DICOMs were converted to Neuroimaging Informatics Technology Initiative (NIfTI) format and stored in a folder structure similar to DICOMs by using dcm2niiX version v1.0.20220720.^23^, ^24^ The number of converted NIfTI files corresponding to separate scans was 59,039. Information about the scanner manufacturer and the used field strength was extracted from the metadata tags.

T1‐weighted scans were selected from NIfTI files and stored according to Brain Imaging Data Structure (BIDS, see Supplementary Text 1) standard simplifying the image analyses.^25^, ^26^ No simple way to automatically identify the MRI sequence type exists for the real‐world clinical data acquired using various protocols and scanners.^27^ Therefore, we developed an algorithm that first determined if the scan was acquired using T1‐weighting (T1) without contrast agent by applying regex search (see Supplementary Text; for inclusion we used “mpr|mprage|t1_se|sag|tra|T1|TFE” and for exclusion “gd”) to the DICOM metadata tag “ProtocolName.” In addition, it was checked that the scan had sufficiently high resolution and coverage of the brain region based on the number of slices (at least 90). If there was more than one potentially suitable T1‐weighted scan without contrast agent from an examination visit, we included a random one of those to the further processing. After the scans were organized into the BIDS, a comprehensive validation script was executed to ensure the integrity and consistency of the data format.^28^

### 2.3 Processing of MRI scans

The T1‐weighted MRI scans were segmented into gray matter (GM) and white matter (WM) and non‐linearly normalized by using default preprocessing of the Computational Anatomy Toolbox CAT12,^29^ Version 8.1, under Matlab 2022b and Statistical Parametric Mapping software SPM12.^30^ The preprocessing utilized the unified segmentation^31^ to remove B0‐inhomogeneities and produce an initial segmentation that was used for (local) intensity scaling and adaptive non‐local means denoising.^32^ An adaptive maximum a posteriori^33^ segmentation with a hidden Markov random field^34^ and partial volume effect model^35^ was used for the final segmentation. For the non‐linear registration to the MNI152Nlin2009cAsym template, the Shooting method^36^ with modulation was used. Finally, neuromorphometrics atlas was used to define the region of interest (ROI) volumes.^37^

CAT12 yields automatic quality control (QC) measures^29^ (supplemental note 4 of reference 29), and the image quality percentage of 68% was considered to be sufficient for the measures from a particular scan to enter the analysis. We arrived at this limit when manually evaluating scans on both sides of the threshold, and by the fact that this limit included nearly all scans in a separate research cohort (ADNI) that were known to be of acceptable quality, as only scans passing the basic QC done by ADNI were considered (see Section 2.5). We additionally performed the MRI volumetry by another pipeline, SynthSeg,^38^ version 2.0, and used a robust regression technique to identify the measures that were not concordant and therefore needed to be removed from the analysis (Figure S2). Briefly, a robust univariate regression, where CAT12 volumes were predictors and the SynthSeg volumes were responses, was performed using MATLAB (robustfit function, bisquare weighting function). We repeated this for all ROIs (hippocampus, ventricles, total gray matter volume). We defined scans as potential outliers if for any of the ROIs the residuals were larger than 8 times the median absolute deviation (MAD) estimate of the standard deviation (SD) of the residuals. We selected 8 times MAD as the threshold by being able to detect clear outliers in scatter plots without identifying many points that did not appear visually as outliers. The scans detected as potential outliers (29 scans) were subjected to visual inspection (18 were removed from the analysis). Although discordance between SynthSeg and CAT12 does not necessarily mean that both are wrong, it provided us a reasoned approach to visually inspect the scans where the two methods clearly provided different results. The SynthSeg was selected as a comparison method due to its robustness to different imaging artifacts.^39^

Finally, there were 2916 processed scans, of which 2434 (83.5%) were qualified to be included into further analysis (Figure 1). Typical reasons for exclusions were that (1) the scan was not T1 weighted; (2) the scan was T1 weighted, but not covering the whole brain region; or (3) there were severe motion artifacts in the T1‐weighted scan. Moreover, because MRI data starting from year 2003 are included, some of the older scans may have been acquired without 3D imaging and with insufficiently low spatial resolution.

In general we aimed for a generalizable pragmatic approach for identifying and processing of brain MRI scans in which a straightforward algorithm is first applied to identify a fairly large proportion of suitable scans using the available metadata, and then QC measures are applied to exclude unsuitable scans. It is possible that a larger number of scans suitable for analysis could be identified by developing a sophisticated protocol with the annotated metadata,^27^ but despite potential value in maximizing the amount of data to be utilized, such algorithms will always be specialized to the available data and may therefore overestimate the pragmatic applicability of RWD.

We considered total gray matter, hippocampus (left and right), and ventricle (left and right lateral and inferior lateral ventricles) volumes as MRI biomarkers, because they have clear established links to dementia/AD, and have been used previously as secondary endpoints in AD‐related randomized controlled intervention trials (e.g.,^2^, ^40^).

### 2.4 Identification of cognitive status from real‐world health data

To determine the cognitive status of the OSTPRE participants at the time of each brain MRI visit, all records potentially reflecting cognitive issues were first identified from the Hospital Discharge Register (1969–1993), the Care Registers for Health and Social Welfare Care (1994‐), the Causes of Death Statistics (1972‐), Register of Primary Health Care Visits (2011‐), Special Reimbursement Register (1964–), and Drug Purchases (1993–) using relevant codes (Table S3). Cognitive status was categorized as no memory complaints (NMCs), subjective memory complaints (SMCs), mild cognitive impairment (MCI), or dementia.

For each MRI visit, the cognitive status was determined based on the visit date versus register code dates. A participant was categorized as having SMC, MCI, or dementia if at least one relevant diagnostic code was recorded in at least one register before the MRI visit date. If none of the relevant codes were recorded before the MRI visit date, the participant was categorized as NMC. If a milder degree of cognitive impairment at the time of the MRI visit was followed by more pronounced impairment within 1 year after the MRI visit, the participant was categorized as having more pronounced impairment at the MRI visit, that is, SMC followed by MCI within 1 year was categorized as MCI, and MCI followed by dementia within 1 year was categorized as dementia.

### 2.5 The ADNI research cohort as reference for the OSTPRE real‐world MRI scans

The ADNI was launched in 2003 as a public–private partnership, led by Principal Investigator Michael W. Weiner, MD. The primary goal has been to test whether serial MRI, positron emission tomography (PET), other biological markers, and clinical and neuropsychological assessment can be combined to measure the progression of MCI and early AD. For up‐to‐date information, see www.adni‐info.org.^41^

ADNI participants were selected to match the OSTPRE cohort by sex, ethnicity, and cognitive status categories at the population level. This was done simply by including all White female ADNI subjects, as the OSTPRE cohort consisted solely of White women. Because ADNI participants were typically younger than OSTPRE participants, we did not match for age and treated age as a covariate in the regression analyses.

For this study, only MRI scans acquired at the ADNI baseline visit were considered. The scans failing the basic ADNI‐QC done at Mayo Clinic were excluded,^42^ and furthermore, the scans receiving image quality percentage less than 68% by CAT12 automatic QC were excluded (3 of 919 scans). We used the same MRI processing pipeline for ADNI as described above for OSTPRE. Cognitive status at the baseline visit was determined by the ADNI Clinical Core based on cognitive assessments, mainly the Mini‐Mental State Examination (MMSE) and Clinical Dementia Rating (CDR). Table S3 describes the ADNI cognitive status categories.

### 2.6 Statistical methods

The associations between the volume measure of interest (total gray matter, hippocampus, ventricles) and cognitive status in OSTPRE and ADNI data were analyzed using a linear regression model. The standardized volume measure was used as a dependent variable in the model, and categories of cognitive status and cohort were the main independent variables. In addition, age, manufacturer, and field strength, as well as total intracranial volume (TIV) were included as independent variables to adjust for variation in background factors. All brain volume measures were standardized to have a mean of zero and SD of 1 to achieve comparable effect sizes. Potential non‐linearity for age and TIV was accounted for by using cubic B‐splines for those terms in the regression model. As there were several MRI scans for some OSTPRE participants, sandwich estimators were used to adjust for clustering by participant. Predictive margins of measures for cognitive status and cohort were used in the comparisons of contrasts of interest (between cognitive status categories within and between OSTPRE and ADNI). Statistical modeling was conducted in R v4.3.2^43^ using package ggeffects v1.5.2^44^ for determining predictive margins and adjusted contrasts. Package dplyr v1.1.4^45^ was used for data preparation and package ggplot2 v3.5.1^46^ for visualization.

## 3 RESULTS

Population characteristics for the two cohorts are shown in Table 1 and Figure S3. For the included 1885 women from the OSTPRE cohort (2434 T1‐weighted MRI scans) there were 916 comparable women in the ADNI cohort (916 T1‐weighted MRI scans).

**TABLE 1:** Population characteristics.

	OSTPRE	ADNI
**Number of T1‐weighted MRI scans (participants)**	2434 (1885)	916 (916)
**Age**, years, mean (SD)	77.9 (SD 4.5)	72.2 (SD 7.2)
**Scanner field strength**		
3 Tesla	426 (17.5%)	647 (70.6%)
1.5 Tesla	2008 (82.5%)	269 (29.4%)
**Scanner manufacturer**		
Siemens	1972 (81.0%)	513 (56.0%)
Philips	442 (18.2%)	153 (16.7%)
GE	0 (0%)	250 (27.3%)
Toshiba	20 (0.8 %)	0 (0%)
**Cognitive status**		
No memory complaints	967 (39.7%)	231 (25.2%)
SMC	479 (19.7%)	158 (17.2%)
MCI	202 (8.3%)	380 (41.5%)
Dementia	786 (32.3%)	147 (16.0%)

Abbreviations: ADNI, Alzheimer's Disease Neuroimaging Initiative; MCI, mild cognitive impairment; OSTPRE, The Kuopio Osteoporosis Risk Factor and Prevention Study; SD, standard deviation; SMC, subjective memory complaints.

**FIGURE 2:**
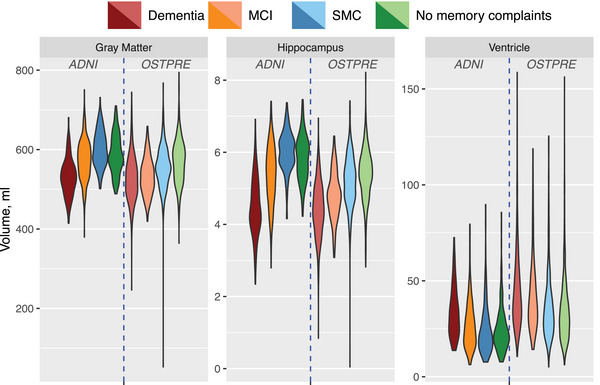
Violin plot of raw values of total gray matter, hippocampus, and ventricle volumes across participants, grouped by four cognitive status categories and cohorts (ADNI and OSTPRE). ADNI, Alzheimer's Disease Neuroimaging Initiative; OSTPRE, The Kuopio Osteoporosis Risk Factor and Prevention Study; MCI, mild cognitive impairment; SMC, subjective memory complaints.

Figure 2 shows the distribution of raw values for total gray matter, hippocampus, and ventricle volume measures in the OSTPRE and ADNI cohorts. The overall pattern across cognitive status categories for these MRI measures was similar between the two cohorts. However, the OSTPRE cohort exhibited a larger variance.

**FIGURE 3:**
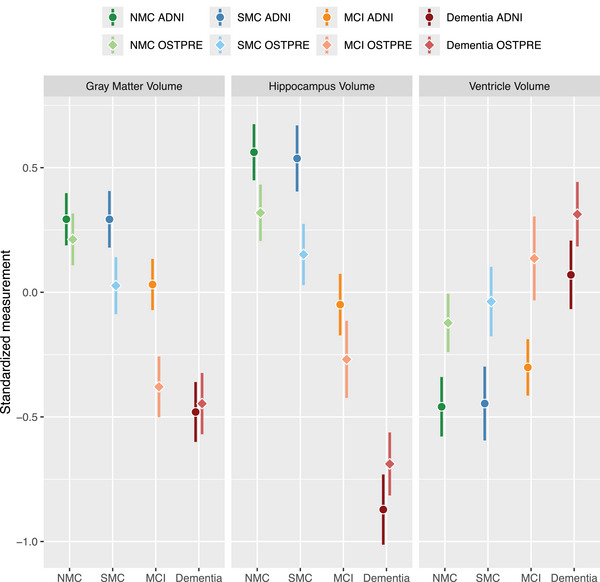
Standardized volume measures with 95% confidence intervals by cohort and cognitive status. ADNI and OSTPRE participants included in analyses are matched by ethnicity, sex, and cognitive status categories. All models are adjusted for age, standardized TIV, manufacturer, and field strength. ADNI, Alzheimer's Disease Neuroimaging Initiative; OSTPRE, The Kuopio Osteoporosis Risk Factor and Prevention Study; MCI, mild cognitive impairment; NMC, no memory complaints; SMC, subjective memory complaints; TIV, total intracranial volume.

In the ADNI dataset, the standardized volume measures were not significantly different between the NMC and SMC cognitive status groups (Table 2, Figure 3). The NMC group was significantly different from both MCI and dementia (*p* ≤ .002), and MCI was different from dementia (*p* < .001). In the OSTPRE dataset, NMC and SMC were different in total gray matter and hippocampus volume (*p* < .001), but not ventricle volume (*p* = .161). NMC was different from MCI and dementia for all volume measures (*p* < .001), whereas MCI was significantly different from dementia for all volume measures except for total gray matter volume (*p* = .242). In both ADNI and OSTPRE, the observed differences were in the expected direction, that is, NMC ≥ SMC > MCI > D for hippocampus and total gray matter volumes, and NMC ≤ SMC < MCI < D for ventricle volume (Figure 3).

**TABLE 2:** Standardized volume measures by cohort and cognitive status categories.

	ADNI		OSTPRE		ADNI–OSTPRE differences	
	Between‐group difference (95% CI)	*p*‐value	Between‐group difference (95% CI)	*p*‐value	Between‐cohort difference (95% CI)	*p*‐value
**Total gray matter volume**						
NMC–SMC	0.00 (−0.10 to 0.10)	0.994	0.19 (0.10 to 0.28)	<0.001	−0.19 (−0.32 to −0.05)	0.006
NMC–MCI	0.26 (0.17 to 0.35)	<0.001	0.59 (0.49 to 0.69)	<0.001	−0.33 (−0.47 to −0.19)	<0.001
NMC–D	0.77 (0.65 to 0.89)	<0.001	0.66 (0.56 to 0.76)	<0.001	0.11 (−0.04 to 0.27)	0.141
MCI–D	0.51 (0.40 to 0.63)	<0.001	0.07 (−0.05 to 0.18)	0.242	0.44 (0.28 to 0.60)	<0.001
**Hippocampus volume**						
NMC–SMC	0.02 (−0.08 to 0.13)	0.629	0.17 (0.08 to 0.26)	<0.001	−0.14 (−0.28 to −0.01)	0.040
NMC–MCI	0.61 (0.51 to 0.71)	<0.001	0.59 (0.46 to 0.72)	<0.001	0.02 (−0.14 to 0.19)	0.779
NMC–D	1.43 (1.29 to 1.58)	<0.001	1.01 (0.92 to 1.10)	<0.001	0.43 (0.26 to 0.59)	<0.001
MCI–D	0.82 (0.67 to 0.97)	<0.001	0.42 (0.28 to 0.56)	<0.001	0.40 (0.20 to 0.60)	<0.001
**Ventricle volume**						
NMC–SMC	−0.01 (−0.14 to 0.11)	0.835	−0.09 (−0.21 to 0.03)	0.161	0.07 (‐0.10 to 0.25)	0.412
NMC–MCI	−0.16 (−0.26 to −0.06)	0.002	−0.26 (−0.41 to −0.11)	<0.001	0.10 (−0.08 to 0.28)	0.273
NMC–D	−0.53 (−0.66 to −0.40)	<0.001	−0.44 (−0.54 to −0.33)	<0.001	−0.09 (−0.26 to 0.07)	0.264
MCI–D	−0.37 (−0.49 to −0.25)	<0.001	−0.18 (−0.33 to −0.02)	0.026	−0.19 (−0.39 to 0.00)	0.053

*Note*: ADNI and OSTPRE participants included in analyses are matched by ethnicity, sex, and cognitive status categories. All models are adjusted for age, standardized TIV, manufacturer, and field strength.

Abbreviations: ADNI, Alzheimer's Disease Neuroimaging Initiative; OSTPRE, The Kuopio Osteoporosis Risk Factor and Prevention Study; CI, confidence interval; D, dementia; MCI, mild cognitive impairment; NMC, no memory complaints; SMC, subjective memory complaints; TIV, total intracranial volume

Overall, similarities and differences in MRI volumetric measures between cognitive status groups were comparable between the ADNI and OSTPRE cohorts (Table 2, Figure 3). NMC–SMC differences in total gray matter and hippocampus volumes were more pronounced in OSTPRE compared with ADNI (0.19 vs 0.00, *p* = .006 and 0.17 vs 0.02, *p* = .040). The NMC–MCI difference in total gray matter volume was also more pronounced in OSTPRE compared with ADNI (0.59 vs 0.26, *p* < .001). The NMC–dementia difference in hippocampus volume was somewhat more pronounced in ADNI compared with OSTPRE (1.43 vs 1.01, *p* < .001). MCI–dementia differences in all volume measures were also more pronounced in ADNI compared with OSTPRE.

**FIGURE 4:**
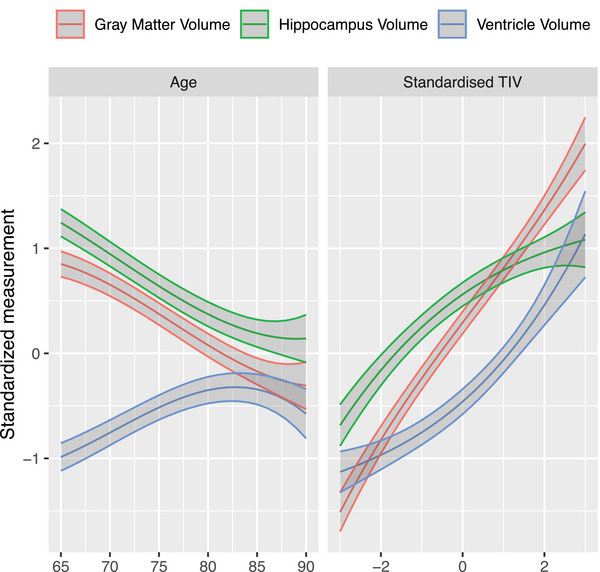
Nonlinear trajectories of MRI measures with 95% confidence intervals by age and standardized TIV in combined cohorts of ADNI and OSTPRE. ADNI, Alzheimer's Disease Neuroimaging Initiative; OSTPRE, The Kuopio Osteoporosis Risk Factor and Prevention Study; MRI, magnetic resonance imaging; TIV, total intracranial volume.

Most brain volumetry studies continue to apply linear adjustment for confounding variables (age, sex, TIV) albeit nonlinear relations between confounding variables and brain volumes have been reliably demonstrated.^47^, ^48^ We addressed the question in the case of real‐world MRI. Figure 4 shows the nonlinear trajectories of MRI measures by age and standardized TIV in a combined cohort of ADNI and OSTPRE (stratified by cohort in Figure S4). Hippocampus and total gray matter volumes decreased with increasing age, but the slope of decline was less pronounced in the oldest old (80+–85+) participants. Similarly, ventricle volume increased with age, with a less pronounced slope of decline in the oldest old. Although all MRI volumes increased with increasing TIV, this increase was not linear for hippocampus and ventricle volumes. The TIV increase was more pronounced than hippocampus volume increase, and less pronounced than ventricle volume increase.

## 4 DISCUSSION

To our knowledge, this is one of the first studies to assess the potential of extracting quantitative biomarkers for AD/dementia from real‐world T1‐weighted brain MRI scans collected over 20 years (2003–2022). We compared three established dementia‐related volumetric measures between four cognitive status groups (normal, subjective complaints, impairments, and dementia) in a large population‐based cohort (OSTPRE) versus a well‐characterized research cohort reference (ADNI). We focused on total gray matter volume, hippocampus volume, and ventricular volume, because they have well‐described links to AD/dementia, and have been previously used as secondary endpoints in AD‐related randomized controlled intervention trials.^2^, ^40^, ^49^, ^50^, ^51^, ^52^ The OSTPRE cohort is the total population of women born in 1932–1941, who lived in the Kuopio region of Eastern Finland in 1989. For these birth years, the Finnish population and the OSTPRE cohort are almost exclusively white Caucasians. Within Finland, the Kuopio region (North Savo) is known to have a higher burden of diseases than the western and southern parts of the country,^53^ but otherwise the cohort represents an unselected group of Finnish women who have been followed through the period of life where changes in cognitive status will start emerging, and therefore it is well suited for observing variations of cognitive status within the population. When brain MRI scans are analyzed, the technical results are unlikely to differ between women and men, but the distribution of cognitive status groups may have gender differences due to, for example, how early or late people seek medical assessment and get referred for brain MRI. Finland has a tax‐funded health care system that provides services for all citizens,^54^ so the general access to care differs from that of some other countries. As the public health care provider collecting the MRI scans covers virtually all residents in the region, OSTPRE cohort can also be considered to be a representative RWD source for MRI scans based on health care needs, without access or cost‐related selection bias. Although Finland and the OSTPRE cohort provide an exceptionally good basis for demonstrating the value of RWD at a general population level, we believe that the results are well generalizable and many RWD sources in other countries will turn out to be similarly usable.

Although the MRI biomarkers were overall aligned between the real‐world and research cohorts, they exhibited significant quantitative variations in their capacity to distinguish between different cognitive status groups. The hippocampus volume separated well all the groups in both cohorts, except for the NMC–SMC comparison in the ADNI cohort. This is a typical finding in research studies.^55^ The significant NMC–SMC difference found in OSTPRE but not ADNI is most likely due to the reliance of the OSTPRE SMC definition on diagnostic codes from RWD, that is, the memory complaints were sufficiently pronounced to require a doctor's referral for examination or at least received doctor's attention during the health care visit for some other reason. In contrast, SMC in ADNI relied on complaints inquired during voluntary research participation. Ventricle volume separated nearly all cognitive status groups in both cohorts, except for NMC–SMC. As expected based on previous findings,^56^ between‐group differences were smaller for ventricle compared with hippocampus volume, indicating that hippocampus volume is a more sensitive dementia‐related marker. Similarly to ventricle volume, total gray matter volume separated nearly all cognitive status groups except for NMC–SMC in both cohorts, and MCI–dementia in OSTPRE. MCI as a diagnostic category is much newer than dementia, that is, older International Classification of Diseases (ICD) versions did not include codes for MCI, and ICD‐10 (introduced in Finland in 1996) has a relatively non‐specific MCI code. Diagnostic criteria for MCI have varied considerably since their first introduction in 1998,^57^ and the timing and consistency of MCI code use in clinical practice is not fully clear. These differences may also be due to the stricter controlled ADNI imaging protocol, or they may reflect the participant selection bias inherent to research cohorts and the specific selection occurring in cohorts tailored for ADRD research. Case–control approaches are particularly prone to overestimating between‐group differences.^58^ In contrast, population‐based cohorts are more likely to reflect the heterogeneity of both populations and MRI methods found in clinical practice.

The distribution of the cognitive status groups showed a higher percentage of dementia cases in the real‐world compared with the research cohort. This is not entirely surprising, since the RWD was based on the presence of a clinical indication for brain MRI referral. Dementia diagnoses in Finnish registers were shown previously to have good accuracy.^59^ Given the limitations of the register‐based MCI definition, the population‐based cohort included significantly fewer individuals with MCI. Newer diagnostic manuals such as ICD‐11 and the Diagnostic and Statistical Manual of Mental Disorder, Fifth Edition (DSM‐5) place more emphasis on this intermediate stage before dementia onset by including a code for mild neurocognitive disorder with more clearly specified criteria.^60^, ^61^ As dementia diseases are increasingly diagnosed at earlier stages, diagnostic criteria evolve with the development of more accessible biomarkers (e.g., blood), and disease‐modifying therapies become available, the distributions of cognitive status groups in population‐based cohorts may also change over time.

To our knowledge, few other studies have focused on extraction of quantitative MRI volume markers from routine MRI scans in the context of ADRD research. Two studies assessed RWD data on MRI scans in the context of computer‐aided diagnosis.^62^, ^63^ They extracted T1‐weighted MRI scans from the data warehouse of 39 French hospitals^63^ and Research Patient Data Registry of a Mass General Brigham system,^62^ respectively. Bottani et al.^63^ demonstrated the difficulty of distinguishing persons with dementia diagnosis from persons without diagnoses suggesting dementia and the presence of brain lesions (only 64.1% balanced accuracy), a task that is considered “easy” with research cohorts. Leming^62^ reported the area under the receiver‐operating characteristic curve (AUC) of 0.82 to separate AD/MCI from healthy controls using convolutional neural network classifier for multimodal clinical brain MRI data. Although the data extraction approach, especially in Bottani et al.,^63^ features many similarities to ours, including the extraction of routine brain MRI scans from the “regional PACS,” our focus of deriving and comparing quantitative MRI parameters differs from the direct application of machine learning algorithm to imaging data classification of these two studies.^62^, ^63^

In the realm of medical research, RWD has emerged as a powerful tool. However, its use in the context of MRI is not without challenges. One of the primary hurdles is the quality of RWD MRI itself. Unlike carefully controlled research settings, clinical environments often produce MRI scans with varying degrees of quality. Factors such as patient movement^64^ and equipment differences (e.g., between different manufacturers) can introduce artifacts, bias, and distortion.^65^ Therefore, to ensure data integrity, researchers must implement (automatic) quality control measures,^14^, ^29^, ^66^ including scripts to check for image quality and standardized protocols for data acquisition. Moreover, the context in which RWD is generated is crucial to understanding its limitations. The referring physicians always have reasons for ordering an MRI examination (and not a computed tomography [CT] examination), and the radiologist who reads the requests may use their own judgement as to the chosen modality and the scanner sequences utilized. Although these situations are mostly covered by mutually accepted protocols, slight deviations can influence the data's relevance and applicability. This study has demonstrated that RWD MRI data have approximately similar quality to that of the data from the largest dementia research program. However, getting the MRI data into use requires multiple steps, which are both legal/contractual (agreements) and technical (availability of tools for mass data export from PACS, pseudonymization, format conversion, computational capacity, and data organization). Therefore, careful planning and utilizing existing standards such as BIDS,^26^ when they exist, is crucial.

We selected CAT12 as our processing pipeline for four reasons: (1) It is an open‐source pipeline with active, ongoing, development; (2) it models partial volume effect, which may be important for volumetry of certain brain structures such as the hippocampus; (3) it contains also a longitudinal processing pipeline, important when assessing changes in MRI markers; and (4) in our experience, it is robust to quality issues in clinical and/or older scans. However, comparing the performance of different processing pipelines with real‐world MRI related to dementia will be an important subject for future study.

In conclusion, although RWD offers immense potential for medical research, its effective utilization requires a nuanced approach. Researchers must navigate methodological challenges, address infrastructural limitations, and carefully consider the context in which RWD is generated. By doing so, they can harness the power of RWD to advance our understanding of diseases and develop more effective treatments. The current study is a crucial first step to ensure that RWD MRI data are usable and can reliably support future RWD studies on topics such as differential diagnosis, disease progression monitoring, or prognosis.

## AUTHOR CONTRIBUTIONS

*Conceptualization: all authors; data acquisition and curation*: Reijo Sund, Sami Väänänen, Jussi Tohka, Elaheh Moradi, Juho Seppänen, and Jani Miettinen; *formal analysis*: Reijo Sund, Juho Seppänen, Jussi Tohka, Elaheh Moradi, and Sami Väänänen; *funding acquisition*: Reijo Sund, Heikki Kröger, Alina Solomon, and Jussi Tohka; *investigation: all authors; methodology: all authors; project management and operational oversight*: Reijo Sund, Alina Solomon, Heikki Kröger, and Jussi Tohka; *scientific oversight*: Reijo Sund, Alina Solomon, Jussi Tohka, Juhana Hakumäki, Heikki Kröger, Heli Koivumaa‐Honkanen, and Toni Rikkonen; *writing – original draft*: Reijo Sund, Alina Solomon, Jussi Tohka, Sami Väänänen, and Juho Seppänen; *writing – review & editing*: all authors.

## CONFLICT OF INTEREST STATEMENT

The authors declare no conflicts of interest. Author disclosures are available in Supporting Information.

## CONSENT STATEMENT

Informed consent was obtained from all human participants.

## CODE AVAILABILITY

Scripts used for the data analyses are available at: https://github.com/rsund/OSTPRE‐brain‐MRI

## Supporting information



Supporting Information

Supporting Information
