# Supplementary material for: Real‐world brain imaging in a population‐based cohort enables accurate markers for dementia
Source: Alzheimers Dement. 2025 Jul 22;21(7):e70227. doi: 10.1002/alz.70227 (PMC12284318; doi:10.1002/alz.70227)
Supplement: Supplementary file 2 — Supporting Information [file ALZ-21-e70227-s001.pdf]

Supplementary materials for the article

Real-world brain imaging in a population-based cohort enables accurate markers for dementia

Reijo Sund, Juho Seppänen, Elaheh Moradi, Sami Väänänen, Jani Miettinen, Juhana Hakumäki, Toni Rikkonen, Heikki Kröger, Heli Koivumaa-Honkanen, Alina Solomon, Jussi Tohka, for the Alzheimer's Disease Neuroimaging Initiative

Supplementary Text 1

PACS (Picture Archiving and Communication System) is a medical image archiving and communication system that uses the DICOM standard to store, manage, and distribute medical images. PACS systems are used in hospitals, clinics, and other healthcare settings to store and retrieve medical images for diagnosis, treatment, and research. PACS systems typically include a server, workstations, and archive storage devices.

DICOM (Digital Imaging and Communication in Medicine) is a standard for storing, transmitting, and communicating medical images and data. It is an international standard that is used by healthcare providers to exchange medical images and data between different imaging modalities, such as X-ray machines, MRI scanners, and CT scanners. DICOM defines a format for encoding image data, as well as a protocol for exchanging images and associated data between devices and networks.

NIFTI (Neuroimaging Informatics Technology Initiative) is a file format for storing and exchanging neuroimaging data. It is widely used in the field of neuroscience to store and share brain images acquired from various modalities, such as MRI, fMRI, and CT scans. NIFTI files contain a standardized representation of the image data, including spatial information, intensity values, and header metadata. This makes it possible to easily share and analyze neuroimaging data across different software platforms and research groups.

BIDS (Brain Imaging Data Structure) is a data format and workflow for organizing and sharing neuroimaging data. It is a standardized way to store and organize neuroimaging data, including neuroimaging scans, metadata, and derived datasets. BIDS is based on the principles of open science and reproducibility, and it aims to make neuroimaging data more accessible and interoperable.

regex (regular expression) is a sequence of characters that defines a search pattern. It is commonly used for string-searching algorithms, "find" or "find and replace" operations on strings, and for input validation. In regex, the vertical bar | is known as the OR operator that makes it possible to match any one of the patterns separated by it. For example, the regex “mpr|T1” will match if either "mpr" or "T1" is found.

Supplementary Figure 1: Number of head MRI examinations for patients over 50 years by year, obtained from the PACS of the Wellbeing services county of North Savo, Kuopio, a large regional public healthcare provider in Eastern Finland. PACS started to serve Kuopio University Hospital in 2003, and district hospitals and primary care units joined the regional PACS between 2006-2011.

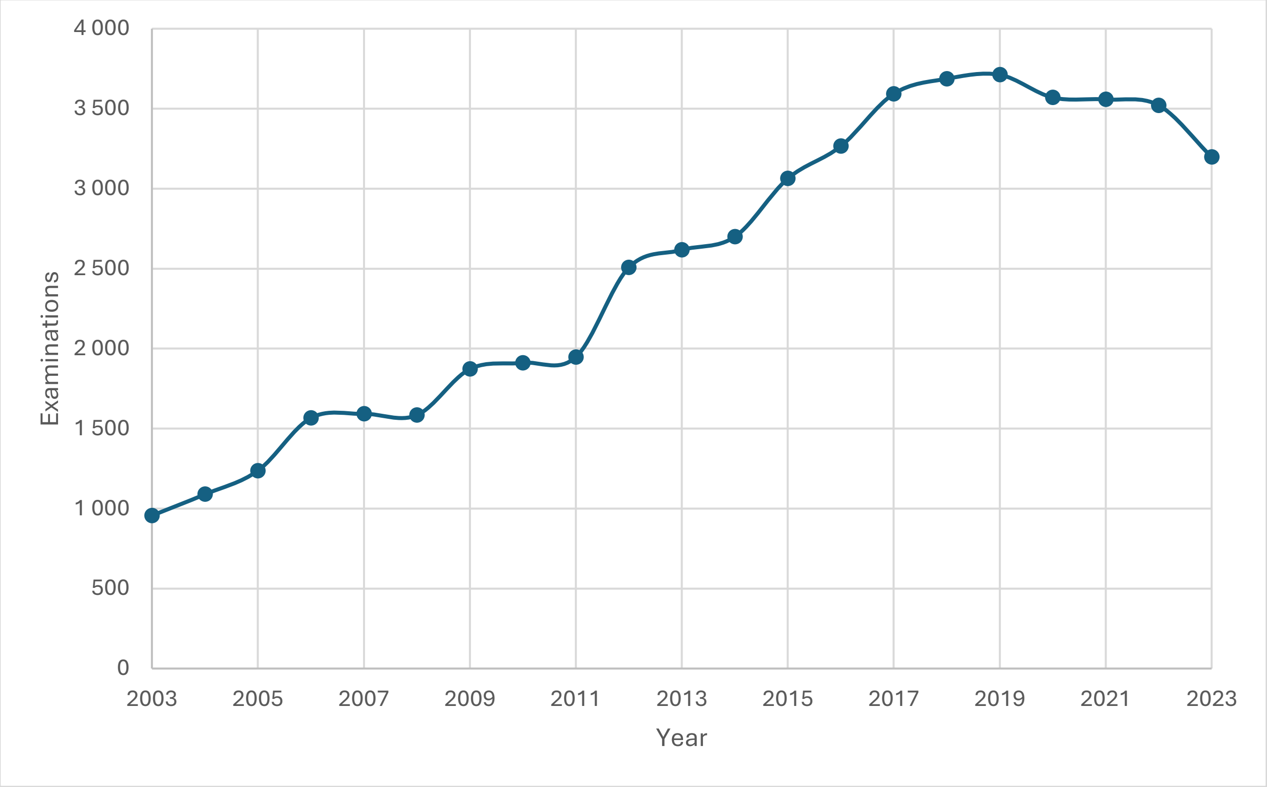

Supplementary Figure 2. CAT12 regional volumes compared to SynthSeg 2.0 volumes with outliers indicated in red. We subjected the scans and segmentations corresponding outlying volumes to further manual quality check.

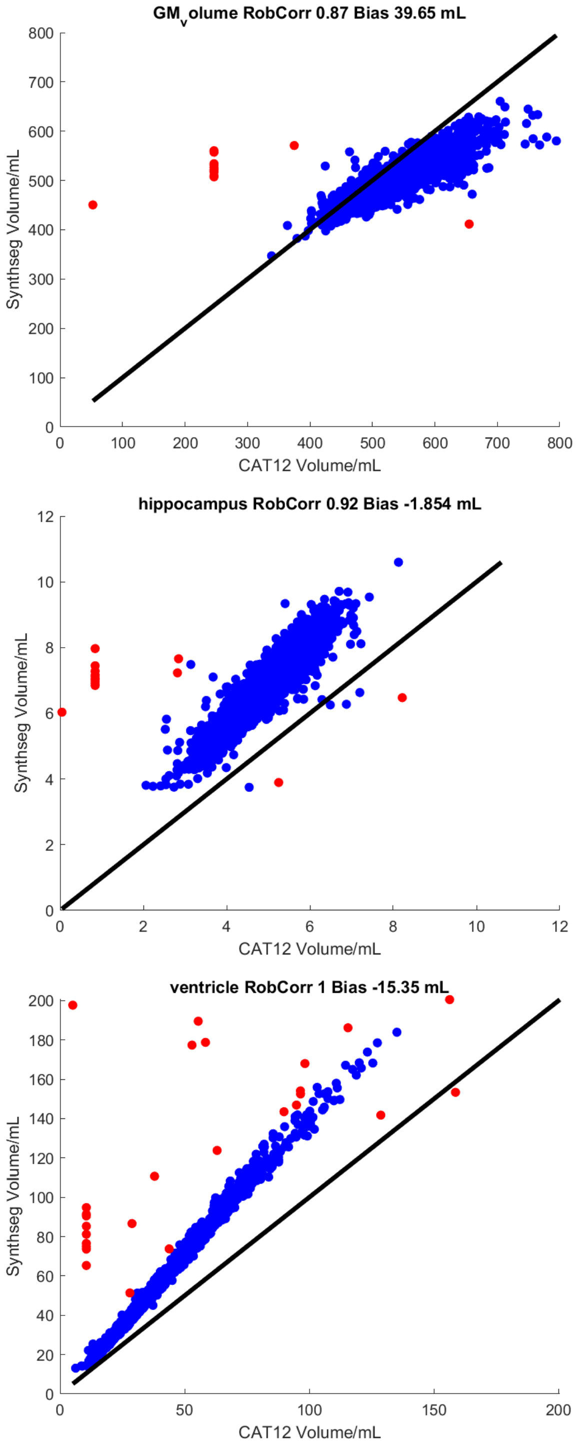

Supplementary Figure 3: Age distribution across diagnostic groups in ADNI and OSTPRE datasets

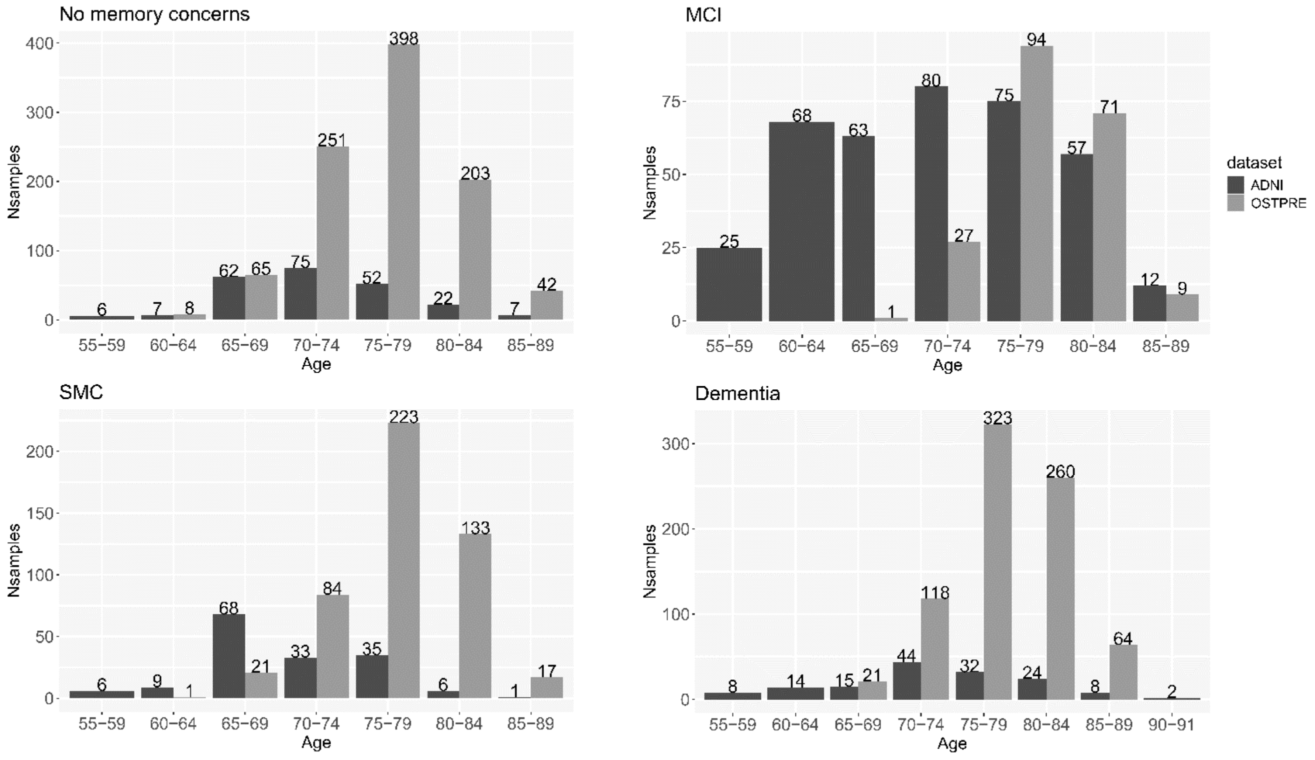

Supplementary Figure 4. Nonlinear trajectories of MRI measures with 95% confidence intervals by age and standardized TIV stratified by cohort (ADNI and OSTPRE). TIV = Total Intracranial Volume, ADNI = Alzheimer's Disease Neuroimaging Initiative, OSTPRE = The Kuopio Osteoporosis Risk Factor and Prevention Study

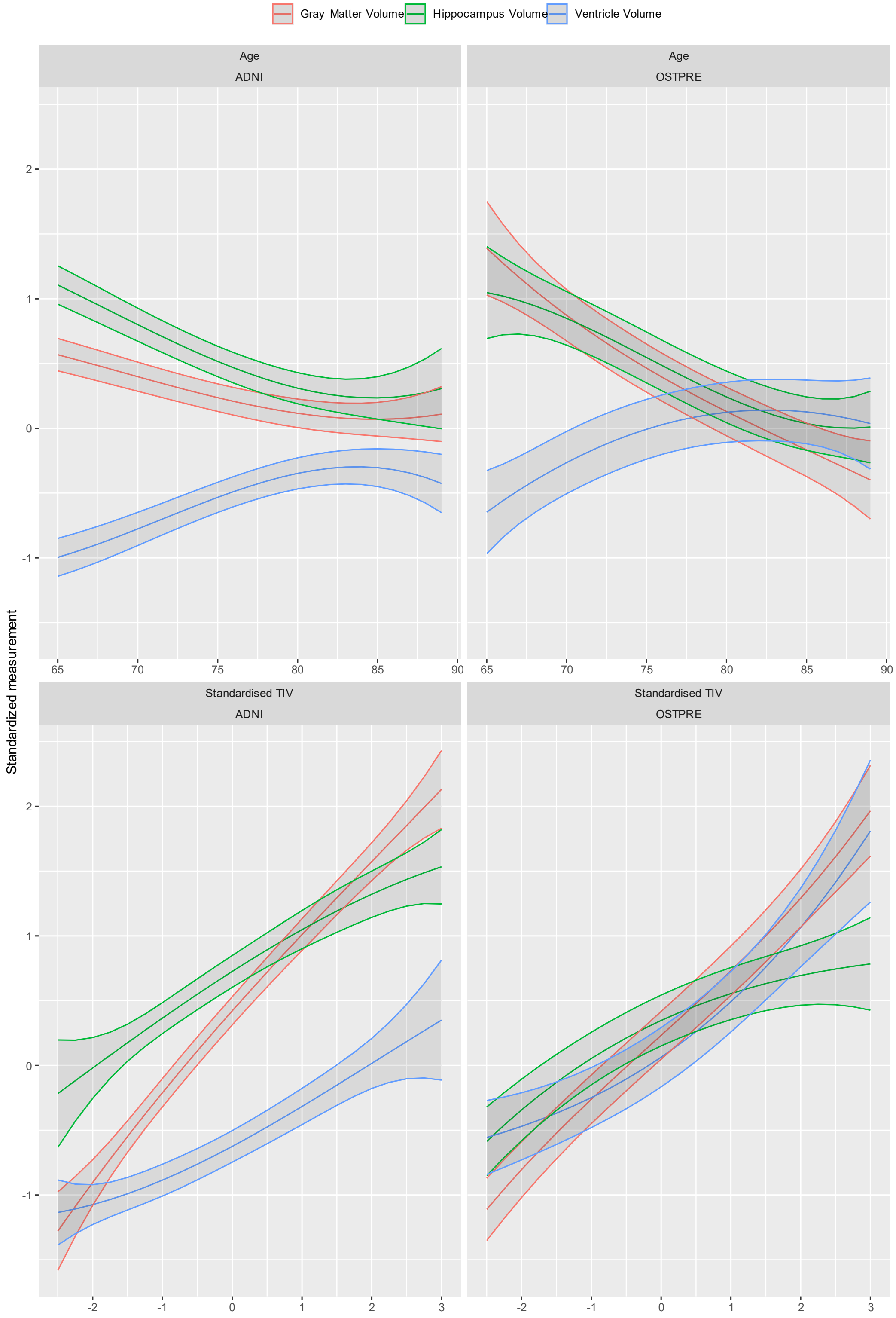

Supplementary Table 1: Number of head MRI examinations for patients over 50 years for each MRI scanner models between years 2003-2023, obtained from the PACS of the Wellbeing services county of North Savo, Kuopio, a large regional public healthcare provider in Eastern Finland. PACS started to serve Kuopio University Hospital in 2003, and district hospitals and primary care units joined the regional PACS between 2006-2011.

Manufacturer	Model	Magnetic field strength	Scanner type	Number of head MRI examinations
Siemens Healthineers	MAGNETOM Avanto	1.5T	Normal	14823
Siemens Healthineers	MAGNETOM Avanto Fit	1.5T	Normal	9411
Philips Medical Systems	Achieva	3T	Normal	9389
Siemens Healthineers	MAGNETOM VISION	1.5T	Normal	3748
Siemens Healthineers	MAGNETOM Sola	1.5T	Normal	3259
Siemens Healthineers	MAGNETOM Aera	1.5T	Normal	2833
GE medical systems	SIGNA Artist	1.5T	Normal	2639
GE medical systems	SIGNA HDxt	1.5T	Mobile	2137
Siemens Healthineers	MAGNETOM Vida	3T	Normal	1956
Siemens Healthineers	MAGNETOM ESSENZA	1.5T	Normal	747
Siemens Healthineers	MAGNETOM VISION plus	1.5T	Normal	415
TOSHIBA	Vantage Elan	1.5T	Normal	216
Siemens Healthineers	MAGNETOM Sola Fit	1.5T	Normal	176
GE medical systems	SIGNA Voyager	1.5T	Mobile	157
GE medical systems	Optima MR360	1.5T	Normal	157
GE medical systems	SIGNA EXCITE	1.5T	Mobile	140
Other or unknown	Scans imported to PACS from other hospitals	Typically 1.5T	Typically normal	577

Supplementary Table 2. Twenty the most common units ordering head MRI examinations for 50+ patients and number of examinations 2003-2023, obtained from the PACS of the Wellbeing services county of North Savo, Kuopio, a large regional public healthcare provider in Eastern Finland. PACS started to serve Kuopio University Hospital in 2003, and district hospitals and primary care units joined the regional PACS between 2006-2011.

Department/Unit	Number of Examinations
Neurology outpatient clinic	15023
Neurology inpatient ward	4730
Neurosurgery inpatient ward	4467
Neurosurgery outpatient clinic	4438
ED clinic	4424
Ophthalmology outpatient clinic	2757
Ear-nose-throat outpatient clinic	2321
Primary care health centre	2025
Geriatric outpatient clinic	1202
Hearing and balance centre	1141
Intensive care inpatient ward	1066
ED ward	1065
Oncology outpatient clinic	851
Endocrinology outpatient clinic	842
Heart outpatient clinic	564
Epilepsy centre	485
Oncology inpatient ward	416
University research scans	411
Internal medicine ward	309
Other	3645

Supplementary Table 3. Description of cognitive status categories in the OSTPRE and ADNI cohorts

**Cognitive status category**	**Definition in OSTPRE cohort**<br>(real-world health data*)	**Definition in ADNI cohort**<br>(research data**)
**Dementia**	*ICD-10 codes:*<br>- F00-F03 Dementia<br>- F1[0-6].73 Dementia due to substance use<br>- F04 Organic amnesic syndrome<br>- F05.1 Delirium superimposed on dementia<br>- G30 Alzheimer's disease<br>*ICD-9 codes:*<br>- 290, 2941, 4378A, 331[1-2] Dementia<br>- 2912A, 2928C Dementia due to substance use<br>- 3310 Alzheimer's disease<br>*ICD-8 codes:*<br>- 290 Dementia<br>*ICPC-2 codes:*<br>- P70 Dementia<br>*ATC codes:*<br>- N06D Anti-dementia drugs<br>*Special drug reimbursement code:*<br>- 307 Alzheimer's disease	MMSE score 20–26 (inclusive), CDR score 0.5 or 1 and meeting the NINCDS/ADRDA criteria for probable AD. The Wechsler Logical Memory II subscale less than 8 (16 or more years of education), less than 4 (8 – 15 years of education), less than 2 (7 or less years of education). Participants with symptoms likely due to non-Alzheimer etiology are excluded.
**MCI**	*ICD-10 codes:*<br>- F06.7 Mild cognitive disorder	MMSE between 24–30 (inclusive), a CDR score of 0.5, and measurable memory deficits based on education-adjusted scores from the Wechsler Memory Scale Logical Memory II: less than 11 for 16 or more years of education, less than 9 for 8-15 years of education, less than 6 for 0-7 years of education Must have a memory concern reported by participant, study partner of clinician, but does not meet the criteria for dementia.
**SMC**	*ICD-10 codes:*<br>- R41 Amnesia<br>*ICD-9 codes:*<br>- 2940 Amnesia<br>*ICPC-2 codes:*<br>- P20 Memory disturbance	MMSE between 24–30 (inclusive), a significant subjective memory concern reported by subject, informant, or clinician, CCI score ≥16 (based on first 12 questions), a CDR of 0, non-depressed, non-MCI, and non-demented, education adjusted scores on delayed recall of one paragraph from Wechsler Memory Scale Logical Memory II (≥9 for 16 or more years of education, ≥5 for 8-15 years of education, ≥3 for 0-7 years of education).
**NMC**	No register recordings of any codes for dementia, MCI, or SMC.	MMSE between 24–30 (inclusive) and a CDR score of 0. These individuals report no memory concerns and show no signs of cognitive decline, remaining fully independent in their daily activities.

ADNI = Alzheimer's Disease Neuroimaging Initiative, OSTPRE = The Kuopio Osteoporosis Risk Factor and Prevention Study, MCI = Mild Cognitive Impairment, SMC = Subjective Memory Complaints, and NMC = No Memory Complaints

* Hospital Discharge Register (1969-1993), the Care Registers for Health and Social Welfare Care (1994-), the Causes of Death Statistics (1972-), Register of Primary Health Care Visits (2011-), Special Reimbursement Register (1964-) and Drug Purchases (1993-)

** https://adni.loni.usc.edu/wp-content/uploads/2024/02/ADNI_General_Procedures_Manual.pdf and https://doi.org/10.3233/JAD-150729
